# Proton beam irradiation stimulates migration and invasion of human U87 malignant glioma cells

**DOI:** 10.1093/jrr/rrt119

**Published:** 2013-11-01

**Authors:** Alexander Zaboronok, Tomonori Isobe, Tetsuya Yamamoto, Eisuke Sato, Kenta Takada, Takeji Sakae, Hideo Tsurushima, Akira Matsumura

**Affiliations:** 1 Department of Neurosurgery, Faculty of Medicine, University of Tsukuba, 1-1-1 Tennodai, Tsukuba, Ibaraki 305-8575, Japan; 2 Department of Radiation Oncology, Faculty of Medicine, University of Tsukuba, 1-1-1 Tennodai, Tsukuba, Ibaraki 305-8575, Japan; 3 Proton Medical Research Center, University of Tsukuba, 1-1-1 Tennodai, Tsukuba, Ibaraki 305-8575, Japan; 4 School of Allied Health Sciences, Kitasato University, 1-15-1 Kitasato, Minami, Sagamihara, Kanagawa 252-0373, Japan

**Keywords:** glioma, proton beam therapy, migration, invasion

## Abstract

Migration and invasion of malignant glioma play a major role in tumor progression and can be increased by low doses of gamma or X-ray irradiation, especially when the migrated tumor cells are located at a distance from the main tumor mass or postoperative cavity and are irradiated in fractions. We studied the influence of proton beam irradiation on migration and invasion of human U87 malignant glioma (U87MG) cells. Irradiation at 4 and 8 Gy increased cell migration by 9.8% (±4, *P* = 0.032) and 11.6% (±6.6, *P* = 0.031) and invasion by 45.1% (±16.5, *P* = 0.04) and 40.5% (±12.7, *P* = 0.041), respectively. After irradiation at 2 and 16 Gy, cell motility did not differ from that at 0 Gy. We determined that an increase in proton beam irradiation dose to over 16 Gy might provide tumor growth control, although additional specific treatment might be necessary to prevent the potentially increased motility of glioma cells during proton beam therapy.

## INTRODUCTION

Malignant gliomas are characterized by fast invasion and long-distance migration of the tumor cells from the main tumor mass or postoperative tumor cavity into the normal brain tissue, and these processes are responsible for tumor recurrence after gross total resection, leading to poor prognosis [1–3]. According to previous reports, low doses of gamma or X-ray irradiation increase tumor cell migration and invasion [4, 5]. A number of molecules, including matrix metalloproteinases (MMPs) [6, 7], integrins [4, 8], and Rac GTPases [9], were found to be responsible for glioma cell motility, and approaches for their inhibition have been studied [10, 11]. However, methods of preventing glioma cell migration and invasion are yet to be found, and, after surgery, radiotherapy remains one of the most commonly used modalities to treat gliomas, including the most malignant, multiform glioblastoma [12–14]. Compared with gamma and X-ray radiotherapy, proton beam therapy (PBT) has been used for decades, mainly to treat hepatocellular carcinoma [15, 16], lung and prostatic cancers [17, 18], and head and neck tumors [19]. In recent years, as an alternative or complement to conventional fractionated X-ray radiotherapy, proton beam irradiation has been proposed for patients with malignant gliomas [14, 20, 21]. In our study, we supposed that small fractions of proton beam irradiation used as a concomitant radiotherapy for patients with malignant gliomas can stimulate migration and invasion of glioma cells, especially when the cells are bordering normal brain tissue. In such cases, even if the lethal dose of ∼ 60 Gy is finally applied to the main tumor mass or the postoperative tumor cavity, with every fraction of radiation, glioma cells located at a distance from the main irradiation field may receive lower doses of radiation, leading to their further movement and to tumor recurrence. Therefore, we examined the effect of proton beam irradiation on migration and invasion of human U87MG cells.

## MATERIALS AND METHODS

### Cell line

Human U87MG cells were obtained from ATCC and cultured at 37^o^C in a humidiﬁed atmosphere of 5% CO_2_ in MEM (Sigma-Aldrich, MO, USA) supplemented with 10% fetal bovine serum (FBS; JRH Nichirei Biosciences, Tokyo, Japan) and 1% penicillin-streptomycin (Sigma-Aldrich, MO, USA).

### Migration and invasion kits

The Oris Universal Cell Migration Assembly kit was purchased from Platypus Technologies, LLC (Fitchburg, WI, USA). Each 96-well plate of the kit contained cell-seeding stoppers (2 mm in diameter) for creating a detection zone at the center of each well. The BD BioCoat Matrigel Invasion Chamber kit was purchased from BD Biosciences (Bedford, MA, USA) and used to study glioma cell invasion.

### Irradiation

The Hitachi proton accelerator at the Proton Medical Research Center of the University of Tsukuba Hospital was used for irradiation. U87MG cells at a concentration of 5 × 10^5^/ml for the migration assay and 2.5 × 10^6^/ml for the invasion assay were placed into transparent plastic tubes in the center of a 105-MeV proton beam (4.5 × 9.5-cm irradiation field) within its spread-out Bragg peak (SOBP). The cells were irradiated at doses of 2, 4, 8 and 16 Gy at 1.36 Gy/min. Nontreated cells were used as controls.

### Cell seeding and analysis

To assess migration, 100 µl of 5 × 10^5^/ml of irradiated cells were seeded into each well around the seeding stopper of the Oris Universal Cell Migration Assembly kit in accordance with the irradiation dose; non-irradiated cells were used as controls. The seeded cells were incubated for 12 h to allow cell attachment to the bottom of the wells. Then, all the wells were examined for proper attachment with an inverted microscope, and the cell stoppers were removed, except for those of the negative controls. The plates were further incubated for 24 h. After incubation, negative control stoppers and media were removed from all wells, and the wells were washed with PBS. For migration assessment, the cells were fixed and stained with a Diff-Quik kit (Sysmex, Kobe, Japan) and scanned, and the cell migration area, representing the sum of the lengths of the tumor cell pathways from the main mass to the center of the well, was calculated.

To assess cellular invasion, 200 µl of 2.5 × 10^6^/ml of irradiated cells were seeded into each BD BioCoat Matrigel Invasion Chamber according to the radiation dose; non-treated cells were seeded at the same concentration and used as controls. The cells were seeded in a medium without FBS, and the 24-h U87MG-conditioned medium was used as a chemoattractant on the other side of the porous membrane. After seeding, the kit was incubated at 37°C in a humidified atmosphere of 95% air:5% CO_2_ for 24 h. After incubation, nonmigrated cells were removed with a cotton swab, and Matrigel™ membranes with 8-µm pores were cut and removed from each chamber. The migrated cells were fixed and stained using the Diff-Quik kit. The membranes were washed, placed on slides, and scanned in at least five fields.

The areas of U87MG cell migration and the numbers of invaded cells were calculated using Biozero software (Keyence, Osaka, Japan) and plotted using Microsoft Excel. All experiments were performed at least three times and statistical significance was calculated by one-way ANOVA.

## RESULTS AND DISCUSSION

**Fig. 1.**
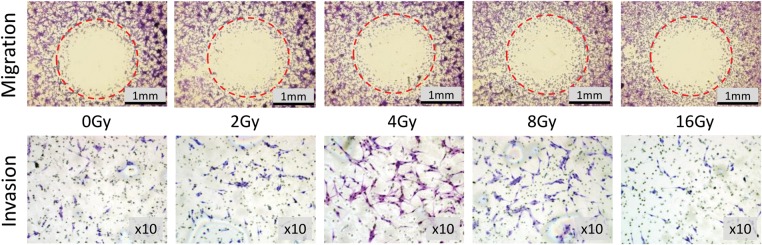
Visualization of U87MG cell migration and invasion. Areas of migration and invading cells after 24 h of incubation (Diff-Quik staining).

**Fig. 2.**
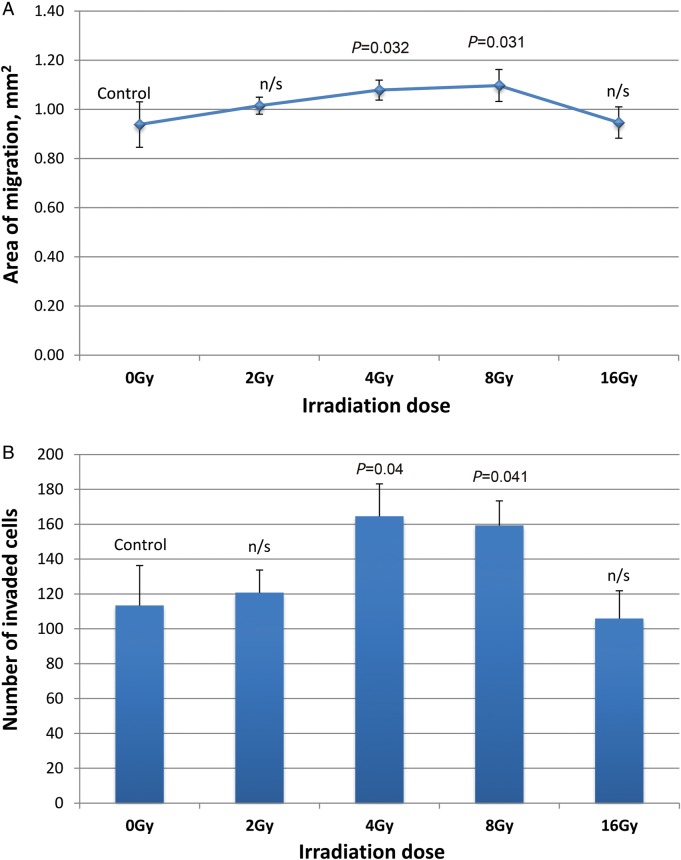
U87MG cell migration (**A**) and invasion (**B**) after 24 h of incubation (means ± SDs, *P*-values by one-way ANOVA).

The U87MG cell migration areas after irradiation and invaded cells are shown in Fig. 1. Quantitative analysis results of both the migration and the invasion assays are presented in Fig. 2. The normal migration area of U87MG cells was determined as 0.938 (±0.093) mm^2^ and the invaded cell number, as 113.34 (±22.96). Proton beam irradiation of 4 Gy and 8 Gy increased U87MG cell migration by 9.8% (±4, *P* = 0.032) and 11.6% (±6.6, *P* = 0.031) and invasion by 45.1% (±16.5, *P* = 0.04) and 40.5% (±12.7, *P* = 0.041), respectively. After irradiation at 2 Gy, glioma cell motility did not significantly differ from that at 0 Gy. Compared with irradiation at 8 Gy, irradiation at 16 Gy significantly decreased migration (*P* = 0.017) and invasion (*P* = 0.002), although the parameters did not differ from those of the control.

Until now, proton beam irradiation has not been as widely used to treat malignant gliomas as X-ray irradiation. Standard X-ray radiotherapy includes 60–61.2 Gy in fractions of 1.8–2 Gy [14, 22]. PBT alone is used as concomitant radiotherapy, but its efficacy is still being analyzed, and clinical trials are ongoing [23]. We previously used 23.1 GyE in 14 fractions (1.65 GyE per fraction) and showed several advantages of this particle radiotherapy over traditional X-ray radiotherapy. In another study, a dose fraction of 1.8 GyE was applied [24]. The dose estimation comes from the relative biological effectiveness (RBE) ratio, which is considered to be 1.1 for PBT, the effect of which is comparable to the effect of 250 keV X-rays [25]. However, it is difficult to predict the RBE in experiments, such as in the current study, and doses in Gy, but not in GyE, were applied. In experiments *in vitro* it is technically very difficult to use ionizing radiation in fractions because living cells are irradiated in the plastic tubes and cannot be stored in the same tubes for more than several hours after the experiment without the results of the experiments being compromised. Therefore, in the current study we used single irradiation doses.

In previous reports on glioma cell migration/invasion, the effects of X-ray tube-based sources [6, 26], photon linear accelerators [5, 21], and gamma rays [4, 27] were presented. In those reports, increase in U87MG cell motility was shown from 1 Gy [5] or 2 Gy [8] of X-rays and from 3 Gy [4] of gamma rays. Wild-Bode *et al*. [4] emphasized the stimulating effects of 1–8 Gy of ^137^Cs gamma rays on U87MG cells with several-fold increased *in vitro* invasion at 3 and 6 Gy associated with elevated MMP-2, MMP-9 and α_v_β_3_ integrin synthesis and with changes in BCL-2 family protein expression; Steinle *et al*. [5] showed BK K^+^ channel activation to be responsible for elevated migration of U87MG cells; and Reiken *et al*. [8] showed that α_v_β_3_ and α_v_β_5_ integrins influenced elevated expression and increase in U87MG motility by around 49.7–191%, depending on the porous membrane coating after 2 Gy of linear accelerator irradiation. The increased migration at 10 Gy was found to be unrelated to the integrins [8]. After proton beam irradiation in our study, we defined the highest rate of migration/invasion at 4 and 8 Gy within the examined dose range. Irradiation at 2 and 16 Gy did not significantly influence U87MG cell motility. Thus, the doses < 2 Gy per fraction used during PBT might be advantageous, as migration and invasion of glioma cells is not stimulated, compared with X-ray radiotherapy. Lack of significant migration and invasion after 16 Gy of proton beam irradiation could indicate the possibility of local tumor growth control with further increase in irradiation dose.

Information on cell survival obtained using such methods as colony-forming assay (CF-assay) might clarify the inhibitory effect of 16 Gy of proton beam irradiation on U87MG migration/invasion. A CF-assay was used in our previous work to show the inhibiting effect of X-ray radiation on U251MG cell survival [28]. Unfortunately, because of their motility, U87MG cells migrated from the original site and failed to form colonies.

## CONCLUSION

In summary, we found a similar tendency of proton beam irradiation to stimulate migration and invasion of human U87MG cells at certain doses, as has previously been demonstrated with X-rays and gamma rays. Nevertheless, using doses of ≤ 2 Gy per fraction during PBT may help to avoid the adverse effects of photon radiotherapy, and may show an advantage over the use of X-rays and gamma rays. Further *in vitro* and *in vivo* migration and invasion studies using proton beam irradiation and several different human glioma cell lines might be of interest to extend and supplement our findings.

## FUNDING

This work was partly supported by a Grant-in-Aid for Scientific Research (C), No. 23592085, from the Japanese Ministry of Education, Culture, Sports, Science and Technology.
